# Reduction in Brain Parenchymal Volume Correlates with Depression and Cognitive Decline in HIV-Positive Males

**DOI:** 10.3390/medicina61040632

**Published:** 2025-03-30

**Authors:** Radmila Perić, Duško Kozić, Snežana Brkić, Dajana Lendak, Jelena Ostojić, Vojislava Bugarski Ignjatović, Jasmina Boban

**Affiliations:** 1 Faculty of Medicine Novi Sad, University of Novi Sad, Hajduk Veljkova 3, 21000 Novi Sad, Serbiajelena.ostojic@mf.uns.ac.rs (J.O.);; 2 Center for Radiology, University Clinical Center of Vojvodina, Hajduk Veljkova 1, 21000 Novi Sad, Serbia; 3 Centre for Diagnostic Imaging, Oncology Institute of Vojvodina, Put dr Goldmana 4, 21204 Sremska Kamenica, Serbia; 4 Clinic for Infectious Diseases, University Clinical Center of Vojvodina, Hajduk Veljkova 1, 21000 Novi Sad, Serbia; 5 Clinic for Neurology, University Clinical Center of Vojvodina, Hajduk Veljkova 1, 21000 Novi Sad, Serbia

**Keywords:** HIV, volumetry, cognitive impairment, depression, neuroimaging

## Abstract

*Background and Objectives*: Human immunodeficiency virus (HIV) has a profound impact on the central nervous system (CNS), contributing to cognitive impairment and depressive symptoms even in individuals receiving combination antiretroviral therapy (cART). This study aimed to investigate the associations between brain parenchymal volumes and neuropsychological outcomes, specifically focusing on cognitive function and depressive symptoms in HIV-positive males. *Materials and Methods*: A total of 48 male participants underwent cognitive assessment using the Mini-Mental State Examination (MMSE), while depressive symptoms were evaluated in 35 participants using the Beck Depression Inventory (BDI). Volumetric brain analysis was conducted through automated imaging software, volBrain (Version 1.0, published on 23 November 2021), ensuring high consistency and accuracy. Statistical analyses included Pearson correlation to identify relationships between brain volumes and neuropsychological outcomes, emphasizing key regions like the basal forebrain and cingulate gyrus. *Results*: Significant trends were observed between basal forebrain volume and MMSE scores, emphasizing the role of this region in cognitive regulation. Additional correlations were found with the anterior and middle cingulate gyri, which are crucial for executive functioning and attentional control. Notably, smaller right basal forebrain volumes were associated with greater depressive symptom severity, suggesting the region’s specific involvement in mood regulation. These findings highlight the dual impact of HIV on cognitive and emotional health, with structural vulnerabilities in key brain regions playing a central role. *Conclusions*: This study underscores the selective vulnerability of certain brain regions, such as the basal forebrain and cingulate gyrus, to HIV-associated neurodegeneration. The results highlight the importance of integrating neuroimaging and neuropsychological assessments in routine clinical care for HIV-positive individuals. The study emphasizes the importance of early detection and targeted interventions to address neuropsychological challenges in this population, with a call for further research in larger and more diverse cohorts.

## 1. Introduction

Human immunodeficiency virus (HIV) is a retrovirus that specifically targets and destroys immune cells expressing the CD4+ receptor, primarily helper T lymphocytes, leading to progressive immunosuppression. This immunosuppressive state renders the body vulnerable to opportunistic infections and malignancies [1,2]. According to recent data from 2023, over 39 million individuals globally are living with HIV, with an estimated 1.3 million new infections occurring annually [3]. While the global prevalence of HIV has been declining over the past decade, effective combination antiretroviral therapies (cART) have played a crucial role in suppressing viral levels to undetectable ranges and reducing transmission risk [3,4]. Despite these advances, HIV presents complex interactions with the immune system, both systemically and within the central nervous system (CNS), resulting in unique neuropathological effects that distinguish CNS involvement from systemic manifestations [1,5].

The CNS becomes vulnerable to HIV early in the course of systemic infection, although CNS-related complications often remain asymptomatic or manifest through nonspecific symptoms [6,7]. In rare cases, acute encephalitis may emerge due to immune responses [8].

While cART effectively suppresses viral replication and reduces viremia in peripheral blood, it does not necessarily decrease immune activation in the brain [9]. Moreover, the widespread use of cART has broadened the spectrum of neuropsychological conditions observed in HIV-positive individuals. These conditions can be grouped primarily into two categories: HIV-associated neurocognitive disorder (HAND) [10] and major depressive disorder [11]. HAND is estimated to affect 30–60% of HIV-positive adults [12,13,14,15,16], with cognitive impairments spanning memory, attention, and executive functions. These deficits often have profound effects on daily functioning and quality of life, underscoring the significant impact of HAND on independence and well-being [13,14].

Depression is another pressing concern in HIV-positive populations, with prevalence rates reaching approximately 31% [17,18]. Compared to the general population, individuals living with HIV are disproportionately affected by depression [19]. Undiagnosed or untreated depression can exacerbate cognitive symptoms, hinder treatment adherence, strain social relationships, and diminish overall quality of life. Furthermore, it may accelerate disease progression and reduce life expectancy in HIV-positive individuals [20].

To evaluate these challenges, tools like the Mini-Mental State Examination (MMSE) and the Beck Depression Inventory (BDI) are commonly used to assess global cognitive function and depressive symptoms, respectively [21,22]. These measures are crucial for understanding the neuropsychological impacts of HIV.

Given the significant burden of cognitive impairment and depression among HIV-positive individuals, investigating the underlying neuroanatomical changes becomes imperative. Understanding how brain volume alterations correlate with neuropsychological outcomes can help guide strategies for cognitive preservation and mental health support [23,24]. This study specifically examines the relationship between brain parenchymal volumes and key neuropsychological factors, focusing on cognitive function and depressive symptoms in HIV-positive individuals.

In our study, only male participants were included to ensure consistency and limit variability in brain volume measurements, recognizing the known influence of gender on brain size and structure [25]. This decision was also based on the epidemiological situation in Serbia, where the male-to-female ratio among newly diagnosed HIV cases is approximately 15:1 [26]. Additionally, including only males eliminated potential hormonal influences on volumetric analyses, providing greater reliability in our findings [27].

This study aimed to investigate the association between brain parenchymal volumes and neuropsychological outcomes, specifically focusing on cognitive function and depressive symptoms in HIV-positive male individuals. We hypothesized that reductions in specific brain regions, such as the anterior cingulate gyrus, are significantly associated with more depressive symptoms and lower cognitive scores.

## 2. Materials and Methods

### 2.1. Participants

All participants in this study were HIV-positive male patients with a controlled viral load who provided written informed consent. All participants were on stable cART (the same regimen for more than 12 months). Inclusion criteria required a confirmed HIV status via PCR without a specific threshold for CD4+ T-cell count. Exclusion criteria included active opportunistic infections, any active neurological disease, documented substance abuse, hepatitis B or C co-infection, detectable lesions on conventional magnetic resonance imaging (MRI), and MRI contraindications.

A total of 48 patients underwent the MMSE as a screening tool for global neurocognitive impairment. The MMSE includes a series of questions and tasks assessing orientation to time and space, attention, calculation, memory, and language skills. Scores range from 0 to 30, where higher scores indicate better cognitive function. Generally, scores are interpreted as follows: 24–30 suggests normal cognitive function, 18–23 indicates mild cognitive impairment, and 0–17 reflects moderate to severe cognitive impairment.

In addition, a total of 35 patients completed the BDI to assess depressive symptoms. The BDI is a 21-item self-report questionnaire that evaluates various aspects of depression, including sadness, loss of interest in activities, changes in appetite and sleep, fatigue, guilt, and concentration difficulties. Responses are scored based on symptom severity, with higher scores indicating greater severity of depressive symptoms. The maximum score is 40, with scores below 10 considered normal (which means the individual does not suffer from depression but has depressive symptoms), scores between 11 and 16 indicating a state of mood disturbance, scores between 17 and 20 suggesting borderline clinical depression, and scores above 20 reflecting clinical depression.

### 2.2. Neuroimaging

All participants underwent conventional MRI on a 3 Tesla MRI scanner (Trio Tim, Siemens, Erlangen, Germany), using a head coil. The imaging protocol included T1-weighted sagittal spin-echo images (TR/TE 440 ms/3.8 ms, slice thickness 5 mm, acquisition time 2:00 min), T2-weighted transverse turbo spin-echo images (TR/TE 5150 ms/105 ms, slice thickness 5 mm, acquisition time 2:57 min), FLAIR transverse images (TR/TE 8000 ms/101 ms, slice thickness 5 mm, acquisition time 3:30 min), diffusion-weighted imaging (DWI) (TR/TE 4100 ms/91 ms, slice thickness 5 mm, acquisition time 2:07 min), and 3D T1-weighted MPR sagittal images (TR/TE 1530 ms/2.97 ms, slice thickness 1 mm, acquisition time 5:12 min).

The imaging data were processed using volBrain software [28], an online platform enabling fully automated volumetric analysis of brain parenchyma from 3D T1MPRAGE images. Version 1.0 of the software, released on 23 November 2021, was utilized in this study. Initial DICOM images were converted to NIFTI format using MRIcron software (version 2.1.57-0, released 30 August 2021) and then uploaded to the volBrain platform. The images were aligned with a standard anatomical template, followed by segmentation of regions of interest using a multi-atlas patch-based approach, referencing a library of manually labeled structures. VolBrain was selected for its efficiency and high accuracy, which make it particularly suited for large-scale studies. While manual segmentation remains the ‘gold standard’, it is time-intensive and prone to subjective errors, such as hand tremors and rater bias. In contrast, volBrain provides reliable and reproducible segmentation, making it an optimal choice for this study [28].

### 2.3. Statistical Analysis

Data analysis was performed using SPSS software (version 23.0). Categorical variables are presented as frequencies and percentages, and continuous variables as medians and interquartile ranges or means and standard deviations, depending on normality. The normality of the distribution was tested using the Kolmogorov–Smirnov test.

For this study, since the primary objective was to assess correlations between brain volumes and cognitive/depression scale scores among HIV-positive patients, Pearson correlation analysis was conducted. A *p*-value of <0.05 was considered statistically significant. For measures that involved multiple comparisons, Bonferroni correction was applied to adjust the significance threshold.To examine potential confounding factors, we performed Pearson correlation analyses to evaluate the relationships between MMSE scores and age, as well as education, and between BDI scores and age, as well as education, while Spearman’s rho analysis was conducted to assess the correlation between MMSE and BDI scores. To evaluate the impact of potential covariates on brain structures for which statistically significant correlations were previously identified, we conducted a Multiple Regression Analysis. The dependent variables were the volumes of these brain structures, while the independent variables included age and years of education.

## 3. Results

### 3.1. Study Population

In this study, a total of 48 HIV-positive male participants were analyzed to investigate the relationship between brain volume changes, cognitive function, and depressive symptoms. The participants had a mean age of 39.26 ± 10.66 years (age range: 25 to 61 years). Cognitive performance was assessed using the MMSE, with an average score of 28.10 ± 1.43 (score range 24–30). Education levels among participants, recorded as the total number of years of formal education, had a mean value of 12.54 ± 2.70 years (range 4–20 years). The average BDI score was 9.24 ± 6.78, indicating that, on average, the group fell within the ‘normal’ range. However, the variability in scores, as reflected by the standard deviation, suggests that while most participants did not suffer from clinical depression, some may have experienced mild depressive symptoms. This highlights the heterogeneity of depressive symptoms within the sample (Table 1).

### 3.2. Correlations Between Key Variables

The results indicate no significant correlations between MMSE and age (*p* = 0.18) or MMSE and education level (*p* = 0.75). Similarly, there were no significant correlations between BDI and age (*p* = 0.42) or BDI and education level (*p* = 0.6). Additionally, the Spearman correlation test revealed no significant relationship between MMSE and BDI scores (*p* = 0.87). Detailed results are provided in the Supplementary File.

### 3.3. Correlations Between Volumes and Neurocognitive Tests

**Figure 1:**
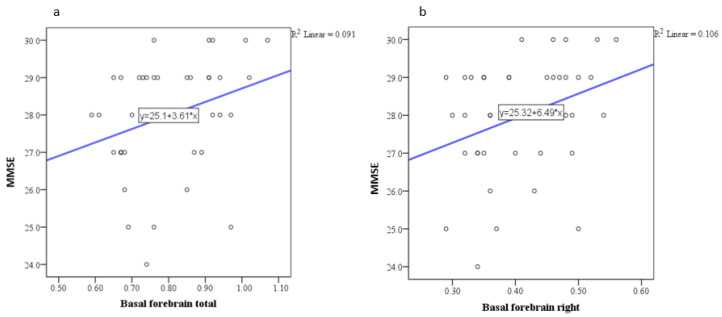
Scatter plot showing the positive correlation between MMSE scores and total (**a**) and right (**b**) basal forebrain volume.

**Figure 2:**
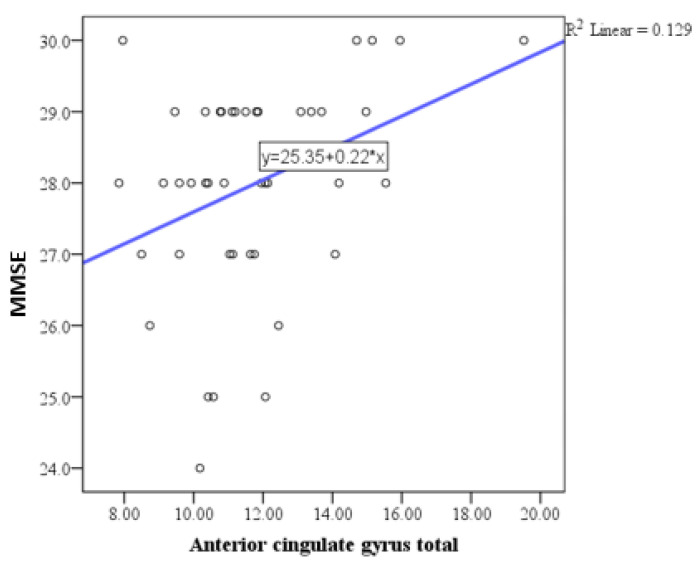
Scatter plot showing the positive correlation between MMSE scores and total anterior cingulate gyrus volumes.

**Figure 3:**
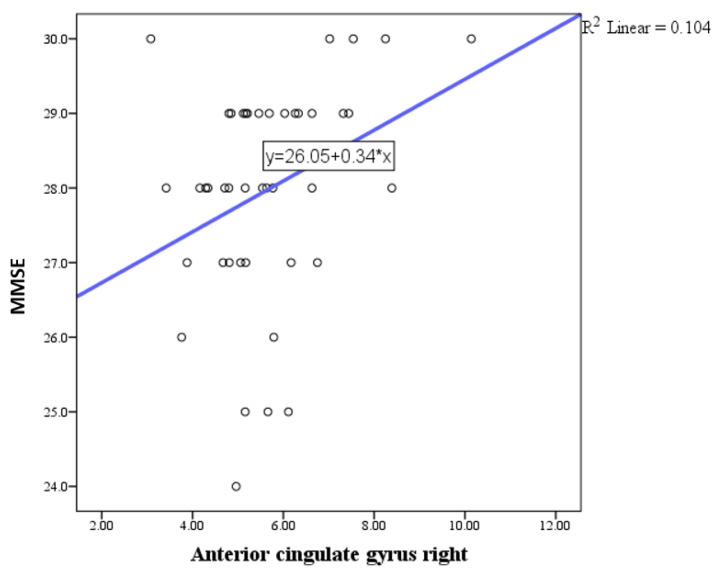
Scatter plot showing the positive correlation between MMSE scores and right anterior cingulate gyrus volumes.

**Figure 4:**
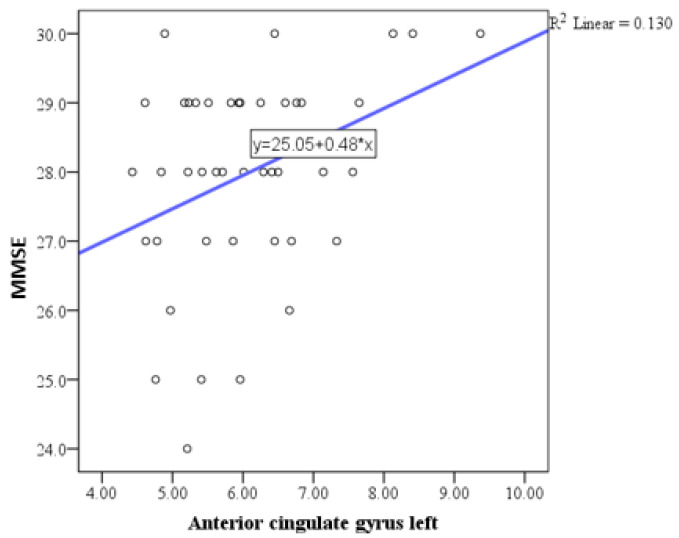
Scatter plot showing the positive correlation between MMSE scores and left anterior cingulate gyrus volumes.

**Figure 5:**
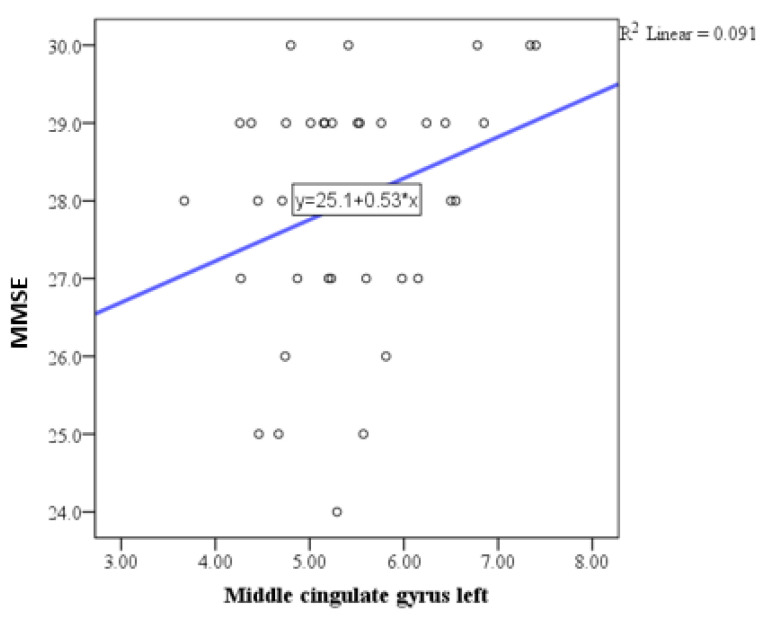
Scatter plot showing the positive correlation between MMSE scores and left middle cingulate gyrus volume.

Pearson correlation analysis indicated a trend toward statistical significance in the correlation between the MMSE and the basal forebrain, particularly on the right side (Figure 1), with a positive correlation of weak to moderate intensity. Similar correlations were observed with the anterior cingulate gyrus (Figure 2, Figure 3 and Figure 4) and the left side of the middle cingulate gyrus (Figure 5). No significant correlations were found between MMSE scores and volumes in other examined brain structures (Table 2).

### 3.4. Correlation of Depression Scale with Volumes

**Figure 6:**
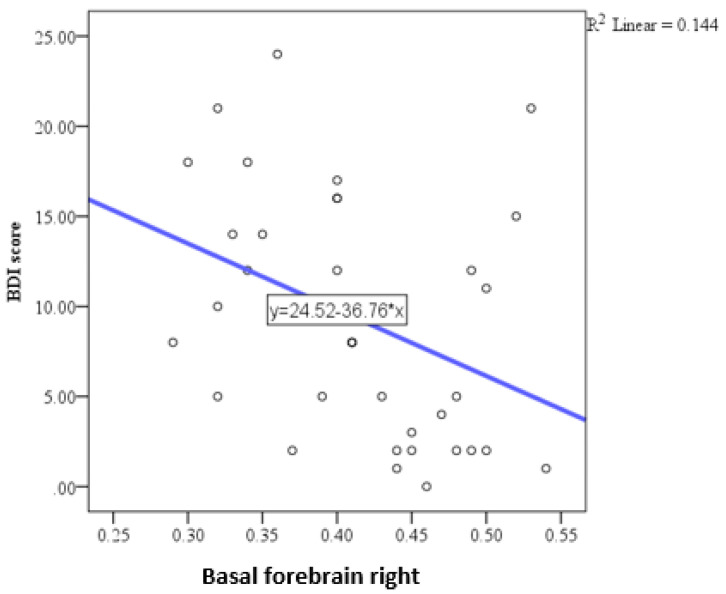
Scatter plot showing the negative correlation between depression scores and right basal forebrain volume.

The depression scale showed a trend toward significant correlation only with the right basal forebrain (Figure 6). This correlation was negative (indicating that a larger volume is associated with lower depression scores) and of moderate intensity (Table 3).

### 3.5. Multiple Regression Analysis Results

The analysis showed that neither age nor years of education significantly predicted the volume of the total basal forebrain (F = 1.132, *p* = 0.326, R = 0.14), the right basal forebrain (F = 2.01, *p* = 0.14, R = 0.19), the total anterior cingulate gyrus (F = 2.51, *p* = 0.09, R = 0.21), the right anterior cingulate gyrus (F = 1.96, *p* = 0.15, R = 0.18), the left anterior cingulate gyrus (F = 2.27, *p* = 0.11, R = 0.2), or the left middle cingulate gyrus (F = 2.39, *p* = 0.09, R = 0.2). Detailed results are provided in the Supplementary File.

## 4. Discussion

The findings of this study highlight the intricate relationship between brain parenchymal volume changes and neuropsychological outcomes in HIV-positive individuals. In this study, Pearson correlation analysis revealed a trend toward statistical significance between MMSE scores and the basal forebrain volume, particularly on the right side, showing a positive correlation of weak to moderate intensity. Additional correlations were observed between MMSE scores and volumes of the anterior and middle cingulate gyri. No significant correlations were identified between MMSE scores and volumes in other brain structures. Regarding depressive symptoms, the depression scale (BDI) demonstrated a trend toward significant correlation exclusively with the right basal forebrain. As mentioned above, we conducted a correlation analysis between MMSE scores and both age and education, as well as BDI scores and both age and education, which did not reveal any significant relationships. The lack of a significant correlation between MMSE scores and education level in our study may reflect the inherent variability in educational standards, which complicates the interpretation of years of schooling as a proxy for educational attainment. These findings are consistent with the study by Boban et al., conducted in our region, which also reported no significant association between MMSE scores and education level [29]. Similarly, no significant correlation was observed between MMSE and BDI scores. Based on this finding, we concluded that MMSE and BDI scores do not interact significantly in our sample, and thus, their inclusion as covariates in the analysis was unnecessary.

The basal forebrain and the cingulum are integral to cognitive and emotional processing, each contributing through distinct but interconnected mechanisms. The basal forebrain, with its cholinergic neurons, plays a central role in modulating cortical processing, dynamic attentional mechanisms, and emotional regulation. Noncholinergic neurons complement this by regulating cortical functions and interacting with cholinergic systems, ensuring adaptability to cognitive challenges [30]. This region is also implicated in depression, where degeneration of cholinergic nuclei leads to cortical cholinergic denervation, stress response dysregulation, and impaired emotional regulation [31]. The cingulum, as a major white matter tract, connects cortical and subcortical regions involved in memory, executive function, and emotional regulation [32]. Together, these structures form a network that is essential for the integration of cognitive and emotional processes, with dysfunction either contributing to depressive symptoms or cognitive decline.

Previous studies have shown that a decline in CD4 lymphocyte levels is associated with an increased risk of depression in HIV-positive individuals. Low CD4 counts can exacerbate neuroinflammation and reduce neurotrophic support, contributing to structural brain changes commonly linked to mood disturbances and cognitive impairment [33,34]. However, in our study, all participants were on cART, which not only increases CD4 lymphocyte levels but also reduces systemic inflammation. This therapeutic effect likely plays a role in mitigating neuroinflammation and its impact on brain structures.

Antidepressant medication, often used to manage depressive symptoms in HIV-positive individuals [6], might have provided an additional neuroprotective effect in our cohort. These medications target key signaling pathways involved in mood regulation and neurogenesis, with clinical trials demonstrating their ability to increase hippocampal volume. For instance, studies have documented volume increases in both the anterior hippocampus [35] and posterior hippocampus [36] following antidepressant treatment. Our findings, which did not reveal significant reductions in hippocampal or amygdalar volumes, contrast with those from studies involving untreated or less effectively treated HIV-positive populations [37]. This discrepancy may be attributed to the combined effects of combination antiretroviral therapy (cART), which restores immune function and reduces inflammation, and antidepressants, which promote neurogenesis and structural resilience. Together, these therapeutic interventions appear to play a crucial role in preserving brain health and mitigating structural changes commonly associated with both cognitive decline and depression in HIV-positive individuals.

Additionally, reductions in brain volume due to aging and neurodegeneration typically begin to manifest around the age of 35, as shown by Perić et al. [38]. Given that the average age of participants in our study was 39.26 ± 10.66 years, the influence of age-related processes on the observed volume reductions cannot be completely neglected. Instead, these reductions seem to be confined to specific brain structures that are particularly vulnerable to the effects of HIV. Despite the neuroprotective effects of cART and antidepressant therapy, these interventions were insufficient to fully protect these regions from HIV-related volume loss, further underscoring the selective impact of HIV-associated neuroinflammation.

Research in frontotemporal dementia (FTD) has demonstrated that basal forebrain atrophy is a key feature of cognitive decline, particularly in tauopathies [39]. Similarly, significant atrophy of the basal forebrain has also been observed in Alzheimer’s disease (AD), where it serves as a key region affected by neurodegeneration [40]. This aligns with our findings, where basal forebrain volume showed a significant correlation with cognitive performance, as measured by MMSE scores, highlighting its role in cognitive regulation. These observations are consistent with prior research emphasizing the basal forebrain’s role in attention, memory, and learning [41]. Moreover, the association between basal forebrain volume and depressive symptoms in our study suggests that this region is also crucial for emotional regulation. Although the underlying mechanisms differ—tau-mediated neurodegeneration in FTD and AD, versus HIV-associated neuroinflammation in our study—the shared involvement of the basal forebrain underscores its vulnerability in neurodegenerative and neuroinflammatory conditions.

Additional correlations were identified with the anterior cingulate gyrus and the middle cingulate gyrus on the left side, which are crucial regions for executive functioning and attentional control [42]. However, no significant correlations were observed between MMSE scores and volumes of other examined brain structures, indicating a selective vulnerability of certain regions to HIV-related neurodegenerative processes.

Depressive symptoms, assessed using the BDI, exhibited a distinct pattern of association. A negative correlation of moderate intensity was found exclusively with the right basal forebrain, indicating that individuals with smaller volumes in this region tend to exhibit higher levels of depressive symptoms. This aligns with evidence linking the basal forebrain to mood regulation through its role in modulating neurotransmitter systems, including acetylcholine and dopamine [43]. The specific involvement of the right basal forebrain may reflect lateralized neural mechanisms underlying emotional processing [44]. The study of Kallianpur et al. reported tending toward a significant negative correlation between depression and volumes of the caudate and the hippocampus, while in other regions that were analyzed (such as nc. accumbens and other subcortical regions) the correlations were not significant [45]. A more recent study, by Bronshteyn et al. [37], presented the negative correlations between the right hippocampal volume and BDI-II scores in a group of HIV-positive people; however, the mean age in the study population was 56.6 years, which is significantly advanced compared to our group, and the effect of aging on the observed reduction in hippocampal volumes cannot be neglected. A very recent study on brain function abnormalities in people living with HIV in a comparable study group failed to present any correlations with gray matter volumes in people living with HIV (PLWH) with anxiety disorder [46]. Resting-state fMRI showed abnormalities in functional connectivity in the anterior cingulated gyrus and amygdala in people with acute HIV, similar to findings in people with depression and without HIV infection, bringing the spotlight more on depression itself [47].

These findings underline the dual impact of HIV on cognitive and emotional health, with structural brain changes in key regions playing a central role. While the basal forebrain appears to serve as a shared substrate for both cognition and mood regulation, the differential correlation patterns for MMSE and BDI scores suggest distinct underlying mechanisms. These could stem from variations in neuroinflammation, synaptic plasticity, or compensatory processes in the affected brain regions [48].

Overall, this study emphasizes the need for early identification of structural brain changes and the development of targeted interventions to mitigate the combined effects of cognitive decline and mood disturbances in HIV-positive individuals.

This study has several limitations. First, the inclusion of only male participants ensures sample homogeneity but limits the generalizability of findings to female HIV-positive individuals, who may exhibit different neuroanatomical and psychological patterns. Second, the relatively small sample size may have reduced the statistical power to detect significant correlations, particularly for weaker associations. One of the limitations of this study is the discrepancy in the number of participants with available MMSE (N = 48) and BDI scores (N = 35). This arose due to the retrospective nature of the study, where logistical challenges and variations in participant compliance during data collection led to incomplete BDI score availability. Additionally, the cross-sectional design prevents conclusions about causal relationships between brain volume changes and neuropsychological outcomes. Although the volBrain software offers efficient and reliable automated segmentation, the absence of manual segmentation—often regarded as the ‘gold standard’ despite its practical challenges—represents a potential limitation in ensuring the highest level of volumetric precision. Finally, while the MMSE and BDI scales were used to assess cognitive function and depressive symptoms, these tools may not fully capture the complexity of cognitive deficits and mood disturbances in HIV-positive individuals. The MMSE, in particular, is limited in detecting subtle cognitive changes, especially in younger populations where high scores are typical. This could reduce the sensitivity of the study to identify early or mild cognitive impairments, potentially impacting the interpretation of results.

Future research should aim to address these limitations by including more diverse and larger cohorts, adopting longitudinal designs to explore causal relationships, and utilizing more comprehensive neuropsychological assessments tailored to different age groups. Additionally, investigating interventions targeting specific brain regions, such as the basal forebrain, could provide valuable insights into mitigating cognitive decline and depressive symptoms in HIV-positive populations. Such approaches will improve our understanding of the interplay between neuroanatomy, cognition, and mood disturbances in this group.

## 5. Conclusions

In conclusion, this study highlights the significant impact of HIV-related structural brain changes on cognitive function and depressive symptoms. The observed correlations between brain parenchymal volumes and neuropsychological measures emphasize the vulnerability of specific brain regions, particularly the basal forebrain and cingulate gyrus, to HIV-associated neurodegeneration. These findings underscore the importance of early detection and targeted interventions to address both cognitive decline and mood disturbances in HIV-positive individuals.

## Figures and Tables

**Table 1:** Study population summary.

Characteristics	Number of Participants	Sex	Age (Years)	MMSE Score	Education Level (Years)	BDI Score	cART
Mean ± SD	48	Males	39.26 ± 10.66	28.10 ± 1.43	12.54 ± 2.70	9.24 ± 6.78	All participants

**Table 2:** Correlation between MMSE scores and brain structure volumes.

Structures		MMSE
Accumbens total	Pearson correlation coefficient (r)	0.090
	P	0.566
	N	48
Accumbens right	Pearson correlation coefficient (r)	0.014
	P	0.928
	N	48
Accumbens left	Pearson correlation coefficient (r)	0.165
	P	0.289
	N	48
Corpus amygdaloideum total	Pearson correlation coefficient (r)	0.119
	P	0.443
	N	48
Corpus amygdaloideum right	Pearson correlation coefficient (r)	0.060
	P	0.703
	N	48
Corpus amygdaloideum left	Pearson correlation coefficient (r)	0.175
	P	0.262
	N	48
Basal forebrain	Pearson correlation coefficient (r)	0.304
	P	0.047
	N	48
Basal forebrain right	Pearson correlation coefficient (r)	0.326
	P	0.033
	N	48
Basal forebrain left	Pearson correlation coefficient (r)	0.214
	P	0.169
	N	48
Nucleus caudatus total	Pearson correlation coefficient (r)	0.186
	P	0.233
	N	48
Nucleus caudatus right	Pearson correlation coefficient (r)	0.230
	P	0.138
	N	48
Nucleus caudatus left	Pearson correlation coefficient (r)	0.142
	P	0.365
	N	48
Hippocampus total	Pearson correlation coefficient (r)	0.173
	P	0.266
	N	48
Hippocampus right	Pearson correlation coefficient (r)	0.138
	P	0.379
	N	48
Hippocampus left	Pearson correlation coefficient (r)	0.202
	P	0.194
	N	48
Globus pallidus total	Pearson correlation coefficient (r)	0.023
	P	0.883
	N	48
Globus pallidus right	Pearson correlation coefficient (r)	0.015
	P	0.923
	N	48
Globus pallidus left	Pearson correlation coefficient (r)	0.027
	P	0.864
	N	48
Putamen total	Pearson correlation coefficient (r)	0.227
	P	0.144
	N	48
Putamen right	Pearson correlation coefficient (r)	0.200
	P	0.199
	N	48
Putamen left	Pearson correlation coefficient (r)	0.251
	P	0.104
	N	48
Thalamus total	Pearson correlation coefficient (r)	0.198
	P	0.209
	N	42
Thalamus right	Pearson correlation coefficient (r)	0.193
	P	0.215
	N	48
Thalamus left	Pearson correlation coefficient (r)	0.203
	P	0.191
	N	48
Ventral diencephalon total	Pearson correlation coefficient (r)	0.174
	P	0.265
	N	48
Ventral diencephalon right	Pearson correlation coefficient (r)	0.179
	P	0.251
	N	48
Ventral diencephalon left	Pearson correlation coefficient (r)	0.155
	P	0.322
	N	48
Anterior cingulate gyrus total	Pearson correlation coefficient (r)	0.359
	P	0.018
	N	48
Anterior cingulate gyrus right	Pearson correlation coefficient (r)	0.323
	P	0.035
	N	48
Anterior cingulate gyrus left	Pearson correlation coefficient (r)	0.361
	P	0.017
	N	48
Middle cingulate gyrus total	Pearson correlation coefficient (r)	0.287
	P	0.063
	N	48
Middle cingulate gyrus right	Pearson correlation coefficient (r)	0.206
	P	0.184
	N	48
Middle cingulate gyrus left	Pearson correlation coefficient (r)	0.301
	P	0.050
	N	48
Posterior cingulate gyrus total	Pearson correlation coefficient (r)	0.264
	P	0.087
	N	48
Posterior cingulate gyrus right	Pearson correlation coefficient (r)	0.218
	P	0.159
	N	48
Posterior cingulate gyrus left	Pearson correlation coefficient (r)	0.275
	P	0.075
	N	48

**Table 3:** Correlation between depression scores and brain structure volumes.

Structures		BDI Depression Scale
Nucleus accumbens total	Pearson correlation coefficient (r)	0.019
	P	0.915
	N	35
Nucleus accumbens right	Pearson correlation coefficient (r)	−0.028
	P	0.875
	N	35
Nucleus accumbens left	Pearson correlation coefficient (r)	0.064
	P	0.714
	N	35
Corpus amygdaloideum total	Pearson correlation coefficient (r)	−0.020
	P	0.909
	N	35
Corpus amygdaloideum right	Pearson correlation coefficient (r)	0.039
	P	0.824
	N	35
Corpus amygdaloideum left	Pearson correlation coefficient (r)	−0.093
	P	0.596
	N	35
Basal forebrain total	Pearson correlation coefficient (r)	−0.323
	P	0.059
	N	35
Basal forebrain right	Pearson correlation coefficient (r)	−0.379
	P	0.025
	N	35
Basal forebrain left	Pearson correlation coefficient (r)	−0.204
	P	0.241
	N	35
Nucleus caudatus total	Pearson correlation coefficient (r)	0.042
	P	0.809
	N	35
Nucleus caudatus right	Pearson correlation coefficient (r)	0.037
	P	0.833
	N	35
Nucleus caudatus left	Pearson correlation coefficient (r)	0.047
	P	0.790
	N	35
Hippocampus total	Pearson correlation coefficient (r)	−0.213
	P	0.219
	N	35
Hippocampus right	Pearson correlation coefficient (r)	−0.182
	P	0.294
	N	35
Hippocampus left	Pearson correlation coefficient (r)	−0.245
	P	0.156
	N	35
Globus pallidus total	Pearson correlation coefficient (r)	0.037
	P	0.833
	N	35
Globus pallidus right	Pearson correlation coefficient (r)	0.018
	P	0.917
	N	35
Globus pallidus left	Pearson correlation coefficient (r)	0.048
	P	0.783
	N	35
Putamen total	Pearson correlation coefficient (r)	−0.122
	P	0.487
	N	35
Putamen right	Pearson correlation coefficient (r)	−0.113
	P	0.516
	N	35
Putamen left	Pearson correlation coefficient (r)	−0.126
	P	0.471
	N	35
Thalamus total	Pearson correlation coefficient (r)	0.012
	P	0.947
	N	35
Thalamus right	Pearson correlation coefficient (r)	0.023
	P	0.897
	N	35
Thalamus left	Pearson correlation coefficient (r)	−0.003
	P	0.985
	N	35
Ventral DC total	Pearson correlation coefficient (r)	−0.085
	P	0.626
	N	35
Ventral DC right	Pearson correlation coefficient (r)	−0.064
	P	0.713
	N	35
Ventral DC left	Pearson correlation coefficient (r)	−0.111
	P	0.526
	N	35
Anterior cingulate gyrus total	Pearson correlation coefficient (r)	0.003
	P	0.985
	N	35
Anterior cingulate gyrus right	Pearson correlation coefficient (r)	−0.080
	P	0.648
	N	35
Anterior cingulate gyrus left	Pearson correlation coefficient (r)	0.106
	P	0.548
	N	35
Middle cingulate gyrustotal	Pearson correlation coefficient (r)	−0.024
	P	0.889
	N	35
Middle cingulate gyrus right	Pearson correlation coefficient (r)	−0.013
	P	0.941
	N	35
Middle cingulate gyrus left	Pearson correlation coefficient (r)	−0.030
	P	0.864
	N	35
Posterior cingulate gyrustotal	Pearson correlation coefficient (r)	0.075
	P	0.669
	N	35
Posterior cingulate gyrus right	Pearson correlation coefficient (r)	0.087
	P	0.620
	N	35
Posterior cingulate gyrus left	Pearson correlation coefficient (r)	0.046
	P	0.794
	N	35

## Data Availability

The original contributions presented in this study are included in the article/Supplementary Materials. Further inquiries can be directed to the corresponding author.

## Supplementary Materials

The following supporting information can be downloaded at https://www.mdpi.com/article/10.3390/medicina61040632/s1. Table S1: Correlation between MMSE and age, and MMSE and education level; Table S2: Correlation between BDI and age, and BDI and education level; Table S3: Correlation between MMSE and BDI scores; Table S4: Results of Multiple Regression Analysis of each of the independent variables.
