# Supplementary material for: Reduction in Brain Parenchymal Volume Correlates with Depression and Cognitive Decline in HIV-Positive Males
Source: Medicina (Kaunas). 2025 Mar 30;61(4):632. doi: 10.3390/medicina61040632 (PMC12028741; doi:10.3390/medicina61040632)
Supplement: Supplementary file 1 [file medicina-61-00632-s001.zip › medicina-3381518-supplementary.pdf]

1.1 Correlation Analysis of Age, Education, MMSE, and BDI

The results indicate no significant correlations between MMSE and age (p = 0.18) or education level (p = 0.75), as shown in Table S1. Similarly, no significant correlations were found between BDI and age (p = 0.42) or education level (p = 0.6), as presented in Table S2.

Table S1. Correlation between MMSE and age, and MMSE and education level

		MMSE	age	Educational level
MMSE score	Pearson Correlation	1	-0.21	0.05
	Sig. (2-tailed)		0.18	0.75
	N	48	48	48

Table S2. Correlation between BDI and age, and BDI and education level

		BDI score	Age	Educational level
BDI score	Pearson Correlation	1	0.14	-0.09
	Sig. (2-tailed)		0.42	0.6
	N	35	35	35

The Spearman correlation test revealed no significant relationship between MMSE and BDI scores p = 0.87, as presented in Table S3.

Table S3. Correlation between MMSE and BDI scores

Correlations				
			BDI_score	MMSE
Spearman's rho	BDI_score	Correlation Coefficient	1.00	-0.03
		Sig. (2-tailed)	.	0.87
		N	35	35
	MMSE score	Correlation Coefficient	-0.03	1.00
		Sig. (2-tailed)	0.87	.
		N	35	48

These findings suggest that neither age nor education significantly influenced MMSE or BDI scores in our sample, nor were MMSE and BDI scores significantly correlated. Consequently, these variables were not included as covariates in subsequent analyses, as they were not confounding factors in our study.

1.2 Multiple Regression Analysis

A multiple regression analysis was conducted to examine the combined influence of age and education level on the volume of the brain structures of interest. The analysis revealed that neither age nor education level, when considered together, significantly predicted the volume of the total basal forebrain (F = 1.132, p = 0.326, R = 0.14), right basal forebrain (F = 2.01, p = 0.14, R = 0.19), total anterior cingulate gyrus (F = 2.51, p = 0.09, R = 0.21), right anterior cingulate gyrus (F = 1.96, p = 0.15, R = 0.18), left anterior cingulate gyrus (F = 2.27, p = 0.11, R = 0.2), or left middle cingulate gyrus (F = 2.39, p = 0.09, R = 0.2).

In addition to examining their combined effect, the individual influence of age and education level on brain structure volumes was analyzed separately as part of a multiple regression analysis. The statistical significance of each independent variable in the multiple regression analysis is presented in Table S4.

Table S4. Results of Multiple Regression Analysis of each of the independent variables

Dependent variable	Independent variable	Beta	Standard error	t value	P value	R^2
Basal forebrain total	Age	-0.08	0.01	-0.79	0.43	0.2
	Education level	0.1	0.00	1.04	0.3	
Basal forebrain the right	Age	-0.11	0.00	-1.11	0.27	0.03
	Education level	0.13	0.00	1.34	0.18	
Anterior cingulate gyrus total	Age	-0.18	0.02	-1.92	0.06	0.04
	Education level	0.6	0.06	0.63	0.53	
Anterior cingulate gyrus right	Age	0.18	-0.02	-1.85	0.07	0.03
	Education level	0.02	0.01	0.21	0.83	
Anterior cingulate gyrus left	Age	0.15	0.01	-1.57	0.12	0.04
	Education level	0.09	0.03	0.97	0.33	
Middle cingulate gyrus left	Age	0.17	0.01	-1.77	0.08	0.04
	Education level	0.08	0.02	0.8	0.42	
